# News and Coming Events

**Published:** 1908-06-27

**Authors:** 


### NEWS AND COMING EVENTS.

Sir William Collins, M.D., M.P., has been re-elected Vice-Chancellor of the University of London for the year 1908-1909.

The Committee for the removal of King's College Hospital to South London have received a cheque for £100 from the Viscountess Hambleden, this being her tenth donation in aid of the fund.

Mr. Isaac Bradley, coroner for Birmingham, has been elected president, and Mr. Walter Schröder, deputy-coroner for Central London, has been re-elected hon. secretary of the Coroners' Society of England and Wales.

The King has approved of the appointment of William Allan Jamieson, Esq., M.D., to be Surgeon to the Royal Company of Archers (King's Body Guard for Scotland), *vice* Surgeon Thomas Annandale, Esq., F.R.C.S., deceased.

The President and Board of Management of the British Home and Hospital for Incurables, Crown Lane, Streatham, will give a garden party at the Home on Tuesday, June 30, 1908, at 3.30 p.m., when the Lord Mayor and Lady Mayoress will be present.

Two Worsley Scholarships for the training of medical missionaries for India will be awarded by King's College, London, in October, 1908. The scholars have free tuition and £20 a year for five years. For further conditions applications should be made to Mr. Walter Smith, secretary of the College.

The governors and medical staff of Guy's Hospital will give a garden party on Wednesday afternoon, July 8, at 3.15 p.m., when the medals and prizes will be distributed to the successful students by Lord Rothschild. The laboratories, museums, college, the Henriette Raphael Nurses' Home, and the wards will be open to inspection from 3 to 5.30 p.m.

An anonymous donor has offered to defray the whole cost—£4,000—of erecting a new out-patients' department for the Royal Portsmouth Hospital. New wings were added to the hospital in commemoration of Queen Victoria's Jubilee and Diamond Jubilee, and new children's wards are being erected by public subscription. It is also now proposed to erect a new nurses' home, which will practically complete the entire rebuilding of the institution.

The Therapeutical and Pharmacological section of the Royal Society of Medicine is arranging an excursion to Kew Gardens for this day, Saturday, June 27. At 11 a.m. there will be a demonstration in the Pharmacological Laboratory of University College by Professor Cushny. After luncheon the party will travel to Kew Gardens by the 3.27 p.m. train from Waterloo, and will be conducted round the Gardens by the Curators.

On June 22 Dr. James Hutchison Stirling, LL.D., F.R.C.S. Ed., the distinguished philosophical writer, celebrated his eighty-eight birthday. He was born in 1820 and took the diploma of the Royal College of Surgeons of Edinburgh in 1842, and from 1843 to 1851 he practised as a surgeon in South Wales. From that time onwards he has devoted himself to philosophy, and has published many important works. Of these, perhaps, the best known is "The Secret of Hegel," first published in 1865, which established its author's reputation as a philosophical thinker of a high order.

The Annual Prize distribution of the Medical School of St. Thomas's Hospital was held in the Governor's Hall of the hospital on Thursday, June 25. Sir Charles Bruce, G.C.M.G., presented the prizes to the successful students.

Sir J. Clifford Allbutt, K.C.B., M.D., Regius Professor of Physics, has been appointed the representative of the University of Cambridge upon the General Medical Council for the ensuing five years.

Surgeon-General Sir Alfred Keogh, K.C.B., K.H.P., Director-General Army Medical Service, has been appointed by the Army Council to be chairman of the newly constituted Advisory Council of the Nursing Service for the general hospitals of the Territorial Force.

The King, on June 22, laid the commemoration-stone of the new hospital and dispensary which is being built at Windsor, and which bears the name "King Edward VII. Hospital." His Majesty was accompanied by the Queen and the Prince and Princess of Wales, and was received by Prince Christian, the president of the hospital. After a short religious service, and the reading of an address by the president, to which the King replied, the stone was duly laid, and their Majesties and the other members of the Royal Family inspected the buildings, with which they expressed their satisfaction. In addition to previous donations the King has lately given £200 towards the building fund of the new hospital. Subscriptions amounting to £12,000 have already been received towards the £20,000 required to complete the work.

The fifth annual meeting of the Queen Alexandra Sanatorium, Davos, was held on June 19 in London, Lord Balfour, the president, being in the chair. The report stated that the sanatorium building is at last in existence. At the laying of the foundation-stone, in August last, by Sir George Bonham, the British Minister to Switzerland, a letter of good wishes was sent on behalf of Queen Alexandra. One of the features of the building is that each of the fifty-eight patients will have a separate room and a private balcony. The president said they were hopeful that in twelve or fifteen months the constructive work of the building would be finished. The council had entered into a contract for the sale of the invalids' home at Davos at a good price. Dr. T. Williams (hon. treasurer) said that the sum in hand amounted to £5,202 and £5,000 would be obtained for the old home. But a further sum of £10,000 would be required.

The annual dinner in aid of the funds of the Royal Hospital for Incurables, Putney Heath, was held on June 18, at the Savoy Hotel. Mr. Herbert J. Allcroft (chairman and treasurer) presided, and the company numbered about 100. The chairman, in proposing "Success to the Royal Hospital for Incurables," said that it was founded by Dr. Reed in 1854 for the permanent care of those who through accident, disease, or deformity were disqualified from performing the duties of life. The hospital, or home, as he preferred to call it, was started with six patients, but at the present time there were 227 inmates and 598 pensioners on the list. This was the oldest institution of the kind in London. Not being a hospital for the cure of people, but an institution for the care of those who were incurable, it was not eligible for a grant from the King's Hospital Fund, the Hospital Sunday Fund, or the Hospital Saturday Fund. Donations and subscriptions were announced by the secretary amounting in all to nearly £2,400.

H.R.H. Princess Henry of Battenberg opened, on June 23, a three days' bazaar at the Shoreditch Town Hall, for the purpose of extinguishing a debt of £8,000 on the Queen's Hospital for Children.

A distinguished audience witnessed the matinée given at the Playhouse on June 23 in aid of the Seamen's Hospital Society, *Dreadnought*, Greenwich. A company of talented amateurs gave a triple dramatic bill, a number of children in sailor costumes sold programmes, and excellent music was provided in the intervals. All the seats were taken, and the programme was much appreciated.

In response to the appeal made for the necessary funds for the memorial to the late Professor Annandale, Professor of Clinical Surgery in the University of Edinburgh, a sum of £231 15s. has already been subscribed. The committee entrusted with carrying out the scheme aim at raising £400. There remains, therefore, a sum of £168 5s. still to be found.

Reuter's Agency announces that a fresh Commission is being organised to proceed to East Africa to study sleeping sickness, its object being to continue the work carried on from 1902 until it was temporarily suspended in 1905, owing to the death in England of Lieut. Tulloch, who contracted sleeping sickness during his researches in Uganda. The new Commission will be in charge of Col. David Bruce, C.B., F.R.S., R.A.M.C., who goes for the second time to East Africa in connection with this work. He will be accompanied by Mrs. Bruce and by Capt. Hamilton and Capt. Bakeman, R.A.M.C., and they will proceed to Lake Victoria, on the northern shores of which the Uganda Protectorate is preparing a laboratory for their use. The Commission will study the natural history of the fly and will inquire into Dr. Koch's theory that crocodiles provide food for the *Glossina palpalis*, and will also investigate the question whether the lower animals harbour the parasites and the exact method by which the fly transfers the parasite. The Commission will leave England on September 25.

A meeting to consider the work of the Metropolitan Provident Medical Association was held recently at 107 Westbourne Terrace, Mr. Harben occupied the chair, and among those present were Viscount Milner, Sir John Tweedy, and Sir William Church. The Chairman said the work of the provident dispensaries in London was very imperfectly understood. The application to medical relief of the principle of helping people to help themselves was probably not more than three-quarters of a century old. The first provident medical association was started in the year 1827 in the south-west of London. Since then about forty had been started at different times, and during the past twenty years about twenty associations had been established in connection with the Metropolitan Provident Medical Association. The objects were to provide good medical treatment for the working classes—including attendance at home—on insurance principles, and to relieve the overcrowded hospital out-patient departments by a system of co-operation. Mr. Charles Trevelyan, M.P., moved a resolution declaring that it was expedient that provident dispensaries working in co-operation with the hospitals, under rules generally approved by the medical profession, should be formed throughout the Metropolis, and that funds for the creation of such dispensaries and their maintenance until they reached a self-supporting position were urgently needed by the Metropolitan Provident Medical Association, which had already established twenty-one branches and now provided medical attendance for considerably more than 30,000 persons.

The Anniversary Festival Dinner of the Metropolitan Hospital, Kingsland Road, N.E., will be held at the Whitehall Rooms, Hôtel Métropole, London, on Monday, July 6, at 7.30 p.m. precisely. The Right Hon. Lord Howard de Walden, chairman of the hospital, will preside.

The eleventh annual dinner of the West London Hospital and Post-Graduate College took place on June 23 at the Trocadero Restaurant, Mr. L. A. Bidwell, surgeon to the hospital and dean of the college, presiding. The toast of "The Imperial Forces" was responded to by Inspector-General James Porter, C.B., Director-General of the Medical Department of the Royal Navy, and by Surgeon-General Sir A. Keogh, Director-General of the Army Medical Department. The latter said that the country relied upon the whole medical profession coming to the aid of the Army in time of war. The corps with which he was connected formed a nucleus round which it was hoped the profession would group itself in a time of national emergency. The Chairman proposed the toast of the evening, and said the hospital was known to be one of the most up-to-date and progressive hospitals in London. The Post-Graduate College had made wonderful progress of late years. Last year 200 joined, and they had passed through the college 1,500 doctors. He referred to the number of naval medical men who spent their leave in study at the college. Mr. G. F. Marshall responded for the hospital, and Lieutenant-Colonel Woolbert, I.M.S., for the college.

In the death of Dr. Bertram Louis Abrahams on June 21 the medical profession, not less than the Jewish community in London, loses a brilliant personality which could ill be spared. Dr. Abrahams had achieved a position as a consulting physician unusual indeed for a man scarcely on the threshold of middle age. The deceased physician held the post of senior assistant physician to the Westminster Hospital; he had been Arris and Gale lecturer before the Royal College of Surgeons; he was Fellow of the Royal College of Physicians, and medical inspector of schools under the London County Council. Dr. Abrahams took the keenest interest in everything connected with Israel in London. He was consulting physician to the Jews' Free School, and presided recently at the dinner of the Maccabæans; he was also largely responsible for the foundation of the Jewish Lads' Brigade, to which he devoted much time and energy. In medical literature he had established a considerable reputation by several articles contributed to Sir W. H. Allchin's "Manual of Medicine," and by numerous other writings in the transactions of various societies and in the medical Press.

On July 1 the congratulations of the whole profession will become due to Sir Henry Pitman on the occasion of his hundredth birthday. Whether in point of professional seniority Sir Henry is the *doyen* of the medical men of this country we do not know, but in respect of age and of attainments there can be few whose record approaches his, even distantly. Sir Henry Pitman took his B.A. at Cambridge in 1831, became M.B. in 1840, and M.D. in the following year. In 1845 he was admitted to the Fellowship of the College of Physicans, of which he was Registrar from 1858 to 1889, retiring with the style of Emeritus Registrar. He was Physician to St. George's Hospital and lecturer on medicine to the Medical School from 1857 to 1866, and is Senior Consulting Physician to that institution. Sir Henry was for many years the representative of the College of Physicians on the General Medical Council, and received the honour of knighthood in 1883. At his present advanced age he retains his faculties practically unimpaired, and has, in fact, continued to act until very recently as one of the auditors of the Lancet Relief Fund.

